# Friction taper stitch welding of a duplex stainless steel

**DOI:** 10.1038/s41598-023-48754-6

**Published:** 2023-12-04

**Authors:** Ram Rapaka, Cleber Rodrigo de Lima Lessa, Guilherme Vieira Braga Lemos, Arlan Pacheco Figueiredo, Buchibabu Vicharapu, Thomas Clarke, Amitava De

**Affiliations:** 1 Indian Institute of Technology Palakkad, Palakkad, Kerala India; 2 Federal Institute of Rio Grande do Sul (IFRS), R. Avelino Antônio de Souza 1730, Caxias do Sul, 95043-700 Brazil; 3 Federal University of Santa Maria (UFSM), Rod. Taufik Germano, 3013, Cachoeira do Sul, 96503-205 Brazil; 4 Physical Metallurgy Laboratory (LAMEF) - PPGE3M/UFRGS, Porto Alegre, Brazil; 5 Indian Institute of Technology Bombay, Mumbai, India

**Keywords:** Engineering, Materials science

## Abstract

Friction taper stitch welding (FTSW) is a novel technique that uses multiple inserts to conceal surface crack in a given substrate. The inserts are rotated and forced to fill the crack as plasticized material, and forge with the substrate in solid-state. The process is well suited for alloys such as duplex stainless steel, which suffers degradation of properties during fusion welding. A detailed experimental and theoretical investigation is presented here on FTSW of a duplex stainless steel (DSS). The experimental results show the presence of a ferrite-rich phase along the interface. The results computed by the numerical process model reveal a direct influence of thermal cycle in the amount of ferrite along the joint interface. The welded joint shows near homogeneous structure and properties similar to those of the substrate.

## Introduction

Friction taper stitch welding (FTSW) involves the use of a series of overlapped tapered inserts to conceal long surface-opening cracks in a substrate^1^. The inserts are rotated and forced to deform and flow as plasticized material into the substrate crack one-by-one with a pre-set overlap. The plasticized insert gets forge welded with the substrate in solid-state. FTSW is suitable for heat-sensitive alloys such as duplex stainless steel (DSS) that experiences a substantive loss of mechanical and corrosion resistance properties during fusion welding^2^. Although FTSW is a potential solid-state joining technique, detailed investigations uncovering the influence of important process conditions on the joint structure and properties especially for duplex stainless steel are absent in the literature.

High energy beam and arc welding processes such as gas tungsten arc welding (GTAW) are currently used in joining of duplex stainless steel (DSS) such as DSS 2205. In comparison to GTAW, laser beam welding (LBW) and electron beam welding (EBW) produced joints with a balanced austenite-ferrite ratio in DSS due to lower heat input and higher cooling rate^3–5^. Muthupandi et al.^6^ reported a substantial loss of the corrosion and mechanical properties due to the formation of secondary austenite in multi-pass GTAW of DSS 2205 (UNS31800). Friction stir welding (FSW) of DSS 2205 provided an austenite to ferrite ratio of around 44/56 and improved joint strength in comparison to that in GTAW process, which resulted in an austenite to ferrite ratio of around (41/59) in the welded joint^7^. Sugimoto et al.^8^ welded a super duplex stainless steel using FSW and reported the presence of brittle intermetallic phases in the weldment. However, attempts to quantify the influence of the intermetallic phases on the joint properties are not reported in detail. For instance, Lessa et al.^9^ studied the friction hydro-pillar processing (FHPP) of DSS 2205 to repair the discontinuities commonly observed in offshore structures, and oil and gas pipelines. The authors could restrict the formation of brittle intermetallic phases within the permissible limits for offshore structures (DNV-RP-F112).

FTSW has evolved from a similar solid-state technique FHPP, which is limited to a single insert. Meinhardt et al.^10^ and Lessa et al.^9^ used FHPP for DSS 2205 and found welded joints with acceptable mechanical and corrosion properties. Kanan et al.^11^ and Landell et al.^12^ reported a fairly homogenous joint structure for FHPP of high carbon steel substrates. Vicharapu et al.^13^ reported good joint properties for FHPP of ASTM A36 steel. FTSW of similar steels also showed acceptable joint structure and mechanical property compared to fusion welded joints^14,15^.

A detailed quantitative investigation on FTSW of a DSS grade is therefore reported in the present work using experimental investigations and a computational process model. The effect of the process condition on the structure and properties of the joint area is presented. The mechanical properties of the joint region are examined by using micro-tensile samples and compared with the base material properties. The computed temperature field is utilized to explain the observed structure and properties of the FTSW joint. In particular, an attempt is made to explain the experimentally measured microhardness distribution based on the numerically computed thermal cycles.

## Methods and materials

**Figure 1:**
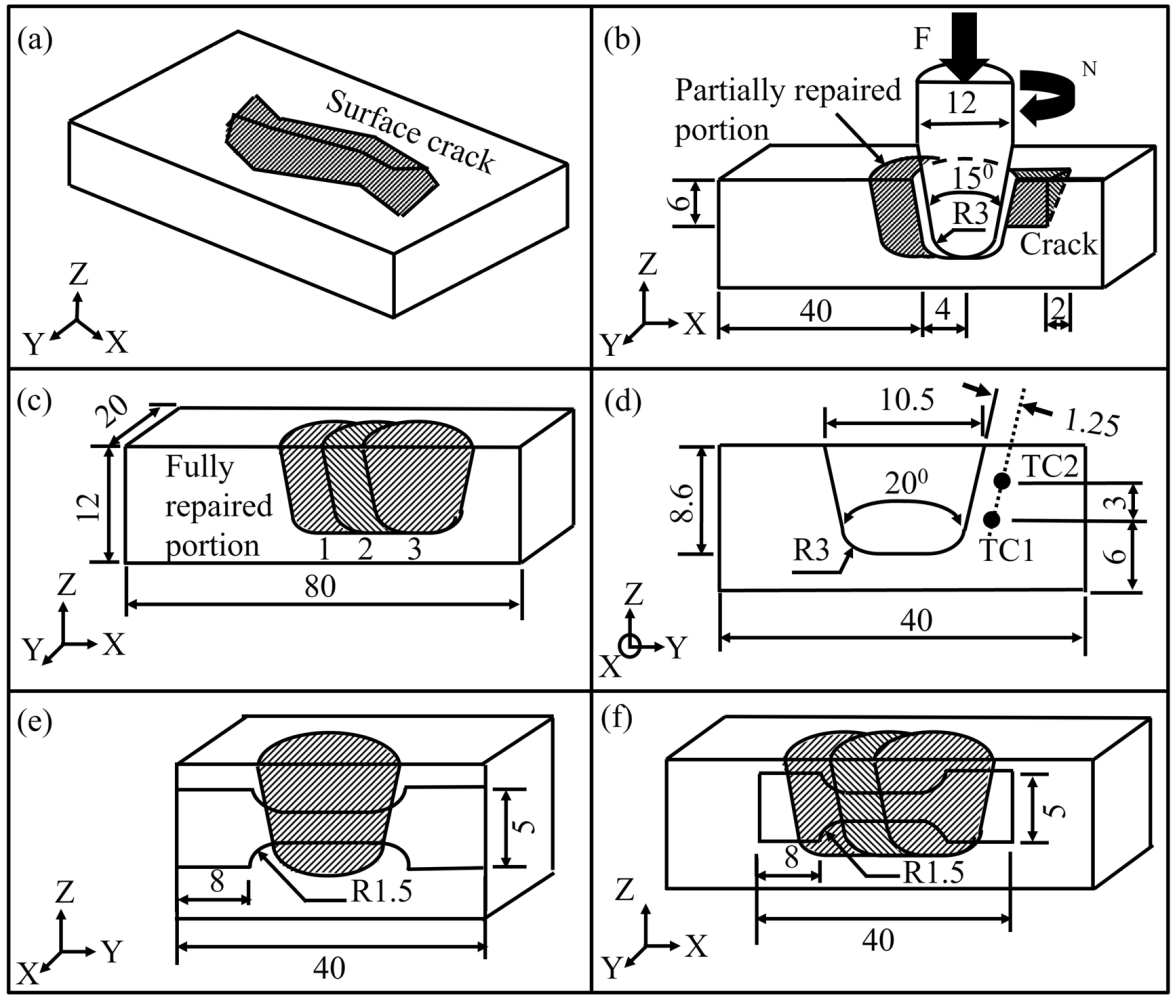
Schematic illustration of (**a**) substrate with a crack open to the surface, (**b**) at beginning of plugging of 2nd insert at a given offset distance, (**c**) fully repaired substrate with three inserts (**d**) substrate cross section (Y–Z plane) depicting two thermocouples TC1 and TC2, and (**e**, **f**) dimensions of the tensile test specimens used to measure tensile properties along the (**e**) transverse, and (**f**) longitudinal directions of the welding.

Figure 1a shows a typical substrate with a surface crack that is first machined to create a continuous groove and subsequently repaired by the FTSW process. The process begins with the drilling of multiple blind holes to expose the root of the entire crack length. Figure 1b shows the insert and the hole with some clearance. The insert is rotated and brought in contact with the base of the root of the crack hole under the action of an axial force. As a result, frictional heating occurs along the interface between the insert and the substrate material at the root of the crack hole leading to thermal softening and plastic flow of insert material inside the crack hole. The rotation of the insert is continued till the plasticized insert material fills the crack hole and excess material comes out of the hole in the form of flash. The rotation of the insert is stopped at this time however, the axial force is continued for a pre-set time to promote forging and coalescence of the plasticized insert material with the substrate. At the end of this time, the axial force is removed, and the flash and the excess insert material are trimmed off from the surface. The requisite times for filling the crack volume by the plasticized insert material and for forging of the plasticized material into the crack volume to yield a joint are finalized by trial experiments. The consequent inserts are introduced with an overlap with the previously welded insert to form a continuous joint thereby filling the entire crack as shown in Fig. 1c.

The experiments are carried out on a built FTSW machine at UFRGS, Brazil^12^ and, using a substrate and inserts of duplex stainless steel (DSS 2205). Figures 1b–d depict the dimensions of the insert, substrate and hole, respectively. A constant overlap distance of 4 mm between two inserts is considered based on preliminary experimental trials. A constant insert rotational speed of 7000 rpm and axial force of 25 kN are used to weld each insert. Figure 1d shows two monitoring locations for the measurement of thermal cycles during FTSW. A separate set of experiments is conducted on a crack free substrate to monitor the thermal cycles during the processing of 1st insert. A set of K-type thermocouples is used for the monitoring of thermal cycles at a sampling rate of 10 Hz.

The sample weld joints are sectioned longitudinally (X–Z) along the direction of the crack for both macro and microstructure analysis. The sectioned weld joint is polished and etched with a Behara modified solution (20 ml HCl, 80 ml deionized water, 1 g K_2_S_2_O_5_ and 2 g of NH_4_HF_2_) for metallurgical characterization. Figures 1e, f show the dimensions of the transverse and longitudinal tensile specimens from the welded region. The transverse specimen contains only one insert within its gauge length as shown in Fig. 1e. The longitudinal specimen is along the length of the original crack and hence, contains three overlapping inserts as shown in Fig. 1f within its gauge length. Four tensile tests are performed at room temperature and the average of the measured results is considered. The microhardness distribution is measured along the joint longitudinal section (X–Z) at three different depths of 2.6 mm, 5.4 mm and 8.0 mm from the substrate top surface. The microhardness is measured at a regular interval of 0.25 mm.

## Theoretical formulation

**Figure 2:**
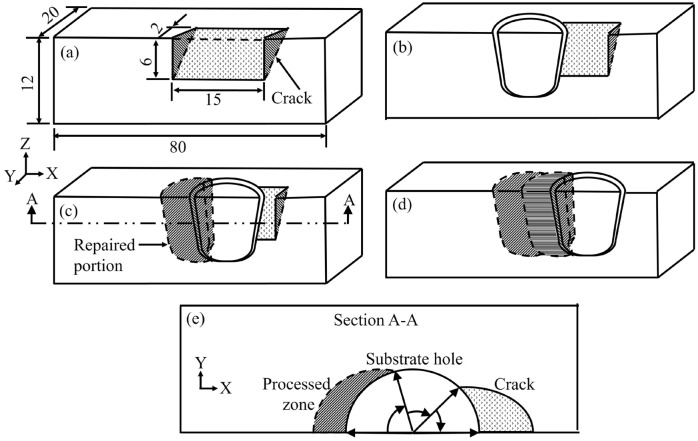
Half symmetric solution domain considered for the modelling of friction taper stitch welding with (**a**) crack, and during the processing of (**b**) 1st insert, (**c**) 2nd insert, and (**d**) 3rd insert. (e) top-view of the section A-A highlights the processed zone and crack opening on either side of the substrate-hole, respectively during the processing of 2nd insert.

A transient heat transfer analysis of FTSW is performed using a commercially available finite element software ABAQUS©/Standard 2020. The solution domain is discretized using 10 node tetrahedron elements (C3D10) with the temperature as the nodal degree of freedom. The analysis considers the rate of frictional heating along the interface around a rotating insert. One half of the actual geometry is considered for the analysis assuming a longitudinal plane of symmetry (x–z plane) passing through the centreline of the longitudinal crack as shown in Fig. 2a^16,17^. The solution domain shown in Fig. 2b includes the pre-machined hole in the substrate, a solid insert and the clearance between the insert and substrate [ref. Figure 1(b)]. The excess insert beyond the substrate top surface is not considered in the model for simplicity. The filling of the clearance between the insert and substrate is modelled by step-wise activation of a set of elements, which are initially defined as inactive. The analytical scheme for the step-wise activation of elements to fill up the clearance region is explained in Appendix-1, supplementary material^11,13^. The joining of subsequent inserts along the length of the crack is modelled in a similar manner as shown in Fig. 2c,d.

The heat transfer for the joining of each insert is modelled using the three-dimensional heat conduction equation

1 $$ \frac{\partial }{\partial x}\left( {k\frac{\partial T}{{\partial x}}} \right) + \frac{\partial }{\partial y}\left( {k\frac{\partial T}{{\partial y}}} \right) + \frac{\partial }{\partial z}\left( {k\frac{\partial T}{{\partial z}}} \right) + \dot{Q} = \rho C\frac{\partial T}{{\partial t}}$$

where *ρ*, *k*, *C*, *T,* and *t* referred to density, thermal conductivity, specific heat, and, temperature and time variables, respectively. The rate of frictional heat generation around the interface of a rotating insert is estimated considering partial sticking and sliding friction as^11,13^

2 $$\dot{Q}=\left[{\eta }_{m}\left(1-\delta \right){\tau }_{Y}+\delta {\mu }_{f}{P}_{N}\right]\left(\omega r\right)\left({A}_{i}/{V}_{i}\right)$$

where *η*_*m*_ is the fractional mechanical work due to sticking friction converted to heat, *δ* is local variation in fractional sliding, *µ*_*f*_ is co-efficient of friction, *r* is the radial distance of a point from the tool axis, *P*_*N*_ is the axial pressure, *ω* is the angular speed of the insert, and *τ*_*Y*_ is the average shear stress of the deformed plasticized material. The average shear stress *τ*_*Y*_ is estimated as a fraction of the flow stress of DSS at a high strain rate of around 100 s^‒1^ from contemporary literature^17–20^. The value of *η*_*m*_ is considered as 0.2 based on reported models on friction stir welding^17–20^. The values of the fractional sliding *δ* and the coefficient of friction *μ*_*f*_ are also considered based on reported models on FSW^17–20^ as

3 $$\delta =-0.026+0.31\times \mathit{exp}(r\omega /1.87); \, {\mu }_{f}=0.51\mathit{exp}(-\delta r\omega )$$

Figure 2e shows the scheme, which is followed to apply the rate of heat generation estimated by Eq. (2). Since every new insert is placed overlapping with the previous one, the interface for the frictional heat generation around a new insert is considered in three parts as θ_R_, θ_H_ and θ_C_. The interface between the new insert and the already welded adjacent insert is depicted as θ_R_, and that between the new insert and the substrate is considered as θ_H_. In the direction of the longitudinal crack, a new insert is likely to face no solid mass along the width of the crack resulting in no heat generation due to friction, and this small region is assumed as θ_C_. The user subroutine DFLUX in ABAQUS is used to apply the varying nature of frictional heat generation for the interface around an insert. An user subroutine UFILM is used to define the convective heat loss from the substrate surface as h(T‒T_a_)^0.25^ and the value of h is considered as 30 W/m^2^ K^0.25^ based on reported models for friction hydro pillar processing^11,13^. Table 1 shows the properties of DSS 2205 for modelling calculations.

**Table 1:** Thermophysical properties and JMA constants of DSS 2205^21,22^.

*ρ* (kg/m^3^), T_S_ (K)	7850, 1650				
k (W/mK)	8.07 + 0.047 T–4.12e−^5^T^2^ + 9.83e−^9^T^3^ for T < 1713 K				
C (J/kgK)	– 72.6 + 1.93 T–1.39e^–3^T^2^ + 3.08e^–7^T^3^ for T < 1713 K				
τ_y_ (MPa)	(916–0.514 × T)/√3 for $\dot{\varepsilon }$ = 10 s^−1^ and T < 1793 K				
	(941–0.528 × T)/√3 for $\dot{\varepsilon }$ = 100 s^-1^ and T < 1793 K				

JMA parameters^23^					
ln(β_o_)	Q (kJ/mol)	R	H_0_ (HV)	H_∞_ (HV)	n
15.7	159.2	8.314	492	253	0.3

T_S_: solidus temperature; other variables are already defined earlier.

The Johnson–Mehl–Avrami (JMA) model is used to predict the microhardness in the SZ, TMAZ and HAZ zones^24,25^. The model uses a linear relation between the hardness and the phase fraction transformed at constant temperature to find the microhardness as

4 $$\mathrm{H}={\mathrm{H}}_{\mathrm{o}}-
\left({\mathrm{H}}_{\mathrm{o}}-{\mathrm{H}}_{\infty }\right)
{\mathrm{e}}^{-{(\upbeta \left(\mathrm{T}\right)t)}^{\mathrm{n}}}\
\mathrm{ where }\,\upbeta \left(\mathrm{T}\right)=\upbeta _{\mathrm{o}}{\mathrm{e}}^{-\frac{\mathrm{Q}}{\mathrm{RT}}}$$

where H_0_ is the maximum hardness due to phase change, H_∞_ is the hardness after a long time, t = ∞, β(T), Q, R and n are the kinetic constant, frequency factor, activation energy, universal gas constant and time exponent, respectively. The JMA constants used for the calculation are shown in Table 1. Further description about the hardness prediction methodology along with the sample calculation are provided in Appendix-2, supplementary information.

### Results and discussion

**Figure 3:**
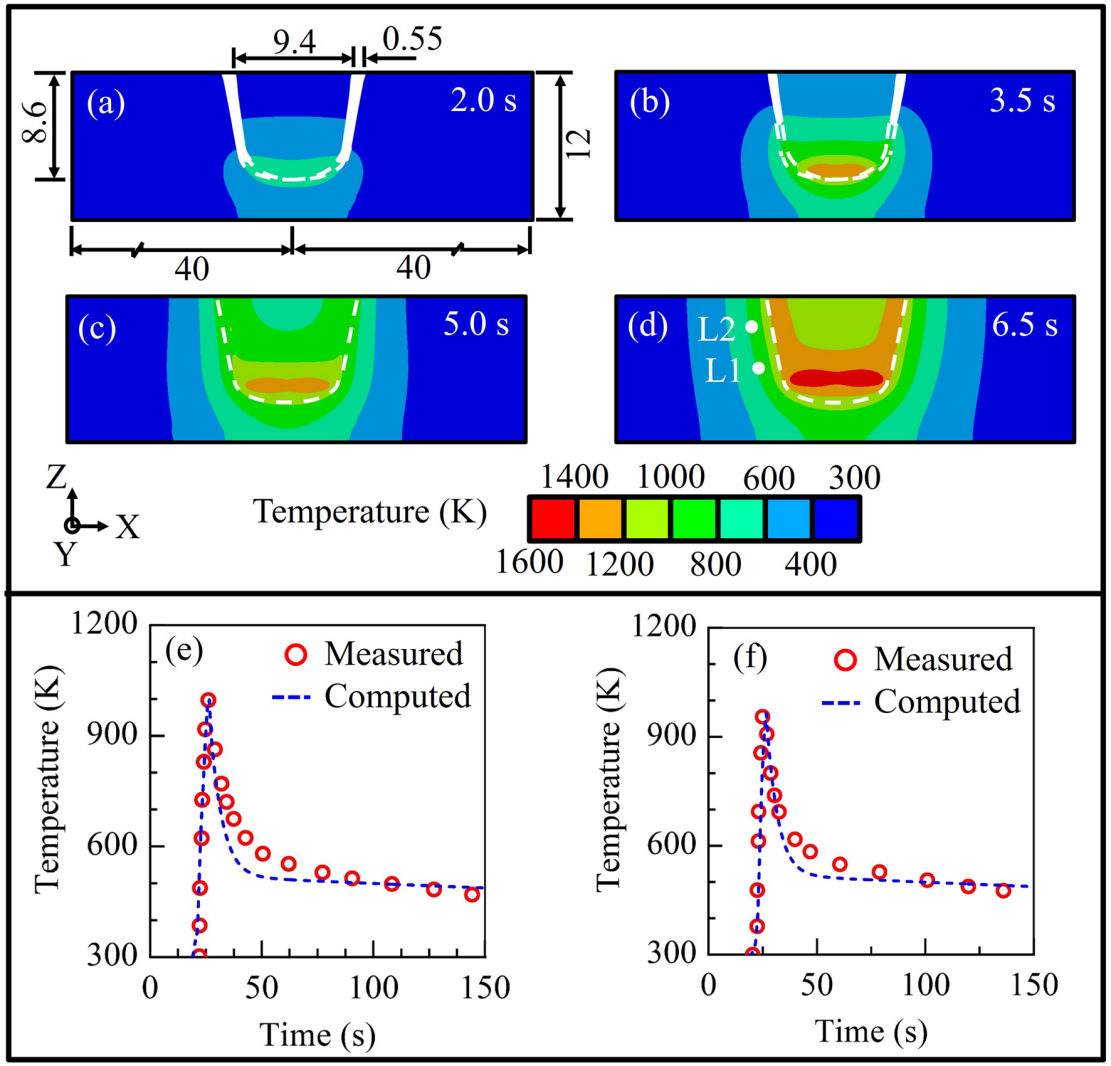
Computed isotherms (**a**–**d**) at a processing time (s) of (**a**) 2.0, (**b**) 3.5, (**c**) 5.0, and (**d**) 6.5. The computed and corresponding measured thermal cycles during the processing of 1st insert at a monitoring location of (**e**) TC1, and (**f**) TC2. The L1 and L2 locations in Fig. (**d**) are identical to the TC1, and TC2, respectively. The white dashed line depicts the original substrate hole boundary.

Figure 3 presents the computed results during the processing of a single insert on a crack free substrate. Figures 3a–d show the computed temperature field at four-time instants of 2.0 s, 3.5 s and 5.0 s, and 6.5 s, respectively as the rotating insert is introduced and, forced to deform plastically and fill the hole. Figure 3a indicates the maximum peak temperature of around 690 K at the interface between the insert tip and the substrate at the end of 2.0 s that is attributed to the friction heat generation as the insert is pressed and rotated against the hole. Figure 3a further shows the clearance between the insert and hole that could be filled up by the plastically deformed insert, where the clearance ensures the joining of insert with hole by ensuring the smoother flow of plasticized material. The filling of the clearance region during the model is accommodated by element birth and death techniques in consecutive time steps as highlighted in section "Theoretical formulation" and Appendix-1, supplementary information. Similar methodology has also been reported for the modelling of FHPP of C-Mn and high carbon steels^11,13^.

Figures 3b,c show the partial filling of the clearance region between the insert and the substrate hole as an insert length of 1.5 mm is introduced and next, the complete filling of the insert when the insert is introduced for a length of around 3.0 mm, respectively. After the complete filling of the substrate hole by the deformed insert material at the end of the 5 s, an additional processing time of 1.5 s is provided for further increase of peak temperature of the interface region as shown in Fig. 3d. The model showed a peak temperature of ~ 1537 K near the insert bottom, which is around ~ 93% of the solidus temperature of the DSS 2205. Kanan et al.^26^ and van Zyl et al.^27^ reported peak temperatures of around 91% and 90% of the substrate materials during FHPP. Higher peak temperature of the interface region aids towards the solid-state coalescence between the substrate and deformed material.

Figure 3e,f show the computed and corresponding measured thermal cycles at two different monitoring locations, TC1 and TC2 (ref. Fig. 1d). The computed and corresponding measured peak temperatures at TC1 and TC2 found to be 997 K and 996 K, and, 965 K and 954 K, respectively. The maximum error between the computed and corresponding measured peak temperature is less than ~ 1%. Both the computed and corresponding measured peak temperatures are found to be slightly higher at TC1 than that at TC2. This is attributed to the rate of friction heat generation along the interface between the tip of the insert and the base of the crack hole under the axial force. Since the monitoring location TC1 is nearer to the interface, the peak temperature at TC1 is slightly higher than that at TC2, which is away from the interface. Similar observations were reported by Kanan et al.^11^ and Landell et al.^12^ during FHPP of AISI 4140 steel. The model is extended further to compute the temperature distribution during the FTSW of three inserts for the real time conditions that are highlighted in Fig. 2.

**Figure 4:**
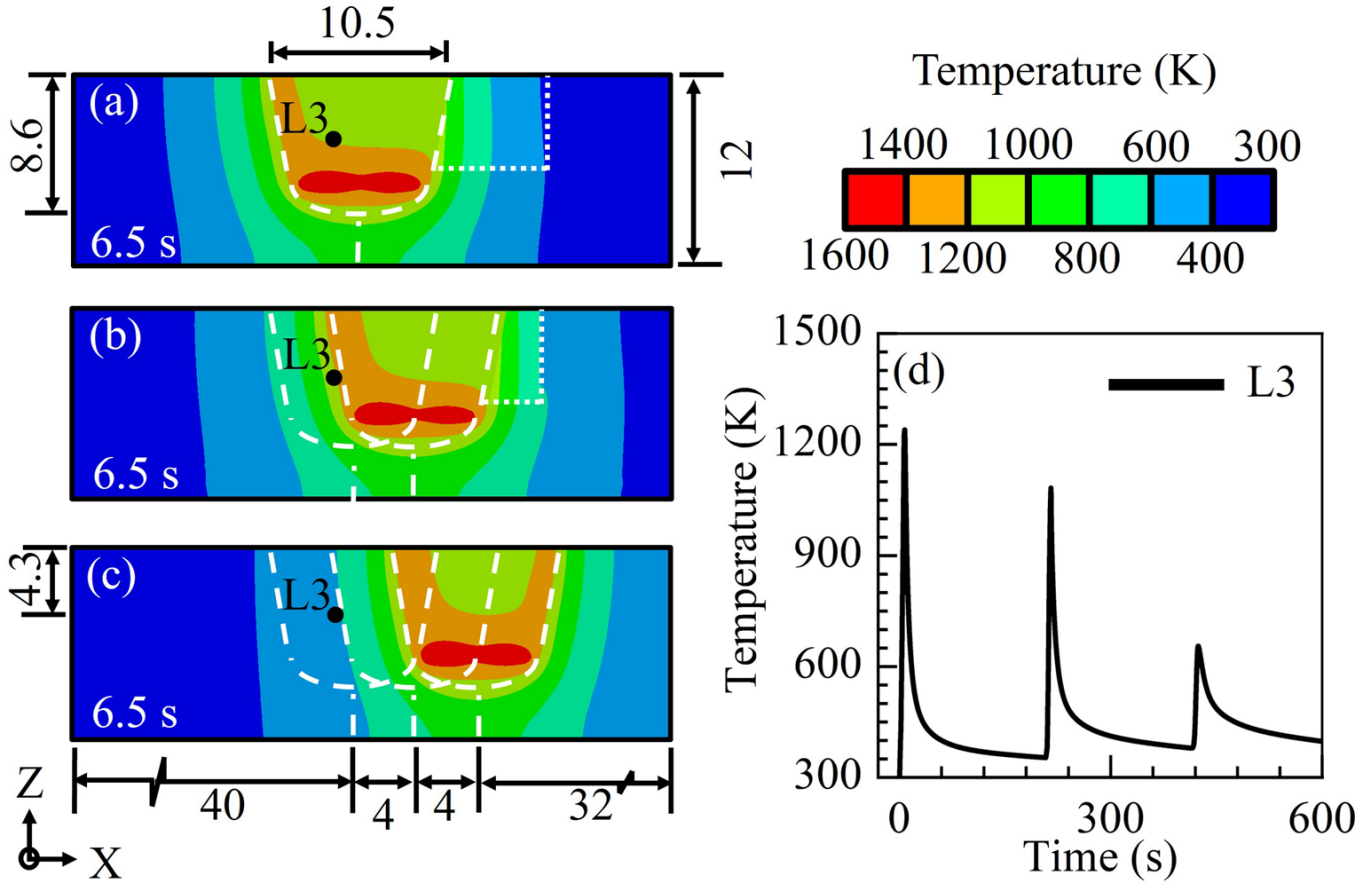
Computed isotherms with crack at the end of the processing stage during the processing of (**a**) 1st insert, (**b**) 2nd insert and (**c**) 3rd insert, and the computed thermal history at location L3. The white dashed and dotted lines depict the original substrate hole and crack boundaries, respectively.

Figure 4a–c shows the computed isotherms after complete filling of the hole by three adjacent overlapped inserts. The computed temperature isotherms in Fig. 4a,b depicts a temperature asymmetry during the welding of the 1st and 2nd inserts, respectively, as frictional heating is absent along the crack width along the length of the crack. A little wider temperature isotherm on the trailing side of the 2nd insert in Fig. 4b is attributed to the frictional heating along the interface between the new insert and the previously welded insert. The computed isotherms in Fig. 4c is at a processing time of 6.5 s and for the introduction of the 3rd insert, which show a near symmetric distribution. As the crack length with the 3rd insert, it experiences frictional heating all around its interface resulting in symmetric temperature isotherms.

Figure 4d presents the computed temperature history at location L3, which is marked with a black dot in Fig. 4a–c. The location is chosen in such a way that this critical point encounters the maximum possible number of thermal cycles with high temperatures. The location L3 typically experiences two potential thermal cycles with peak temperatures exceeding the phase transformation temperatures above 1073 K. Likewise, the model can be used to compute the temperature history at any given spatial location of the solution domain. The computed temperature distribution is further utilized to compute the hardness at any given location of the solution domain. The computed values are validated with the corresponding experimentally measured results.

**Figure 5:**
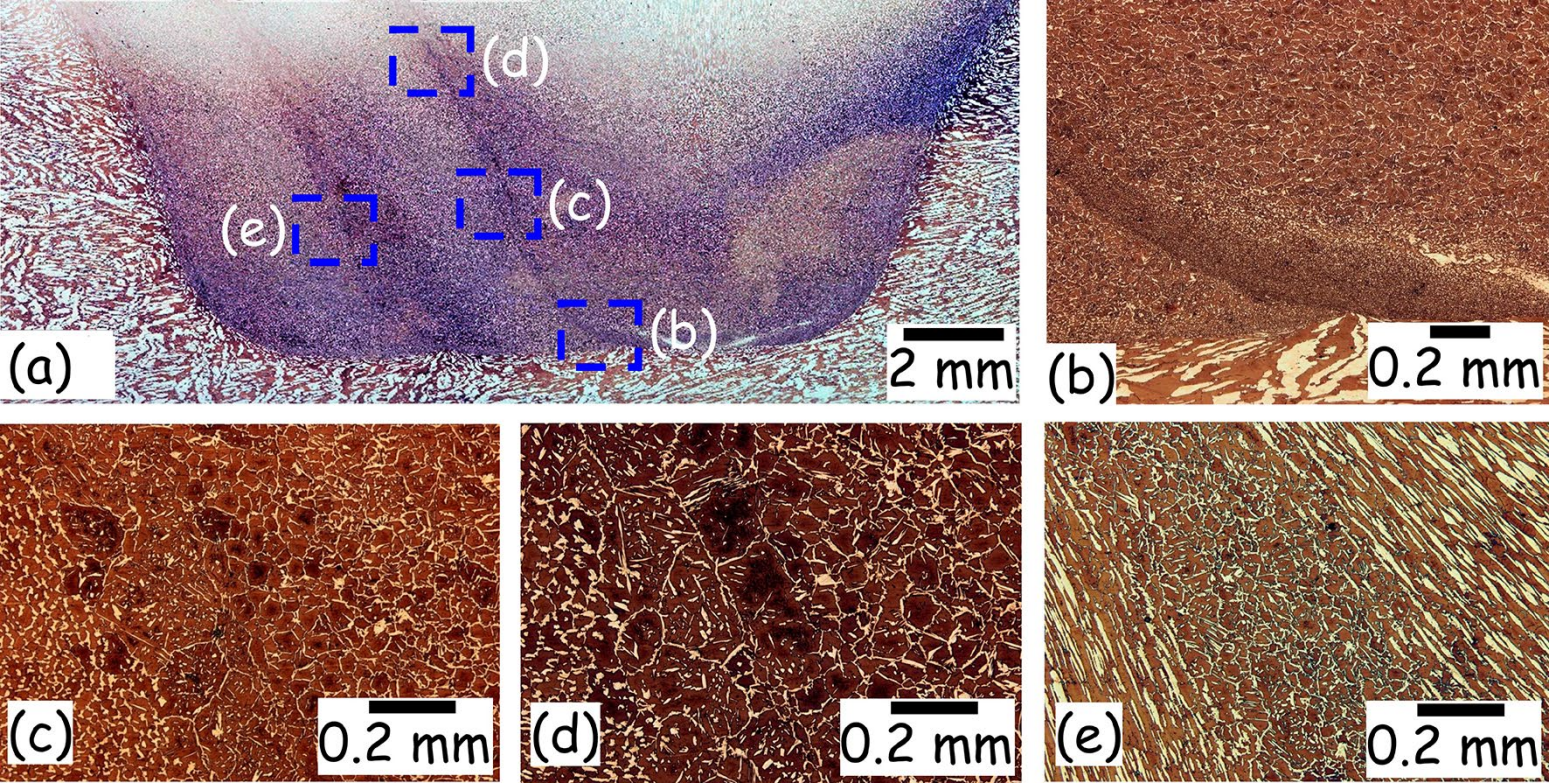
(**a**) Welded joint macrograph in the longitudinal direction of crack, (**b**–**e**) microstructures of the weld joint cross-sections depicting different weld zones.

Figure 5a shows a macrograph of the FTSW joint, in which no surface or volumetric defects (i.e. cracks, voids, and lack of bonding) are seen. Figure 5b depicts a nearly equal proportion of austenite and ferrite phases below the interface between insert and base of the crack hole. In contrast, the insert portion in Fig. 5b contains mostly ferrite, which is attributed to high peak temperatures experienced within the insert (see Fig. 4a). Pissanti et al.^28^ and Zhang et al.^29^ have also reported the presence of chiefly ferrite phases in similar alloys at high working temperature. Additionally, a microduplex microstructure is also present close to the tip of the insert. Figure 5c shows slightly lower concentration of austenite in the insert because of the remained high heat input that favors the ferrite formation. Moreover, Fig. 5d depicts a coarser grain structure (around ~ 1.1 µm) than what is seen in Fig. 5b,c (where the grain size is approximately 0.34 and 0.68 µm, respectively) since the FTSW is much more intense at the tip, thus forming a fine-grained microstructure as noted in the marked locations (b, c). During FTSW, the microstructure is usually elongated, as shown next to the interface in Fig. 5e, but after this solid-state process ends, an equiaxial microstructure is formed due to the remaining heat. In contrast, Fig. 5a presents nearly unaffected microstructure towards the top surface in the centre of the insert. Finally, the defect free joint is evaluated by tensile and microhardness tests as highlighted in Section 2 and Fig. 1e,f.

**Figure 6:**
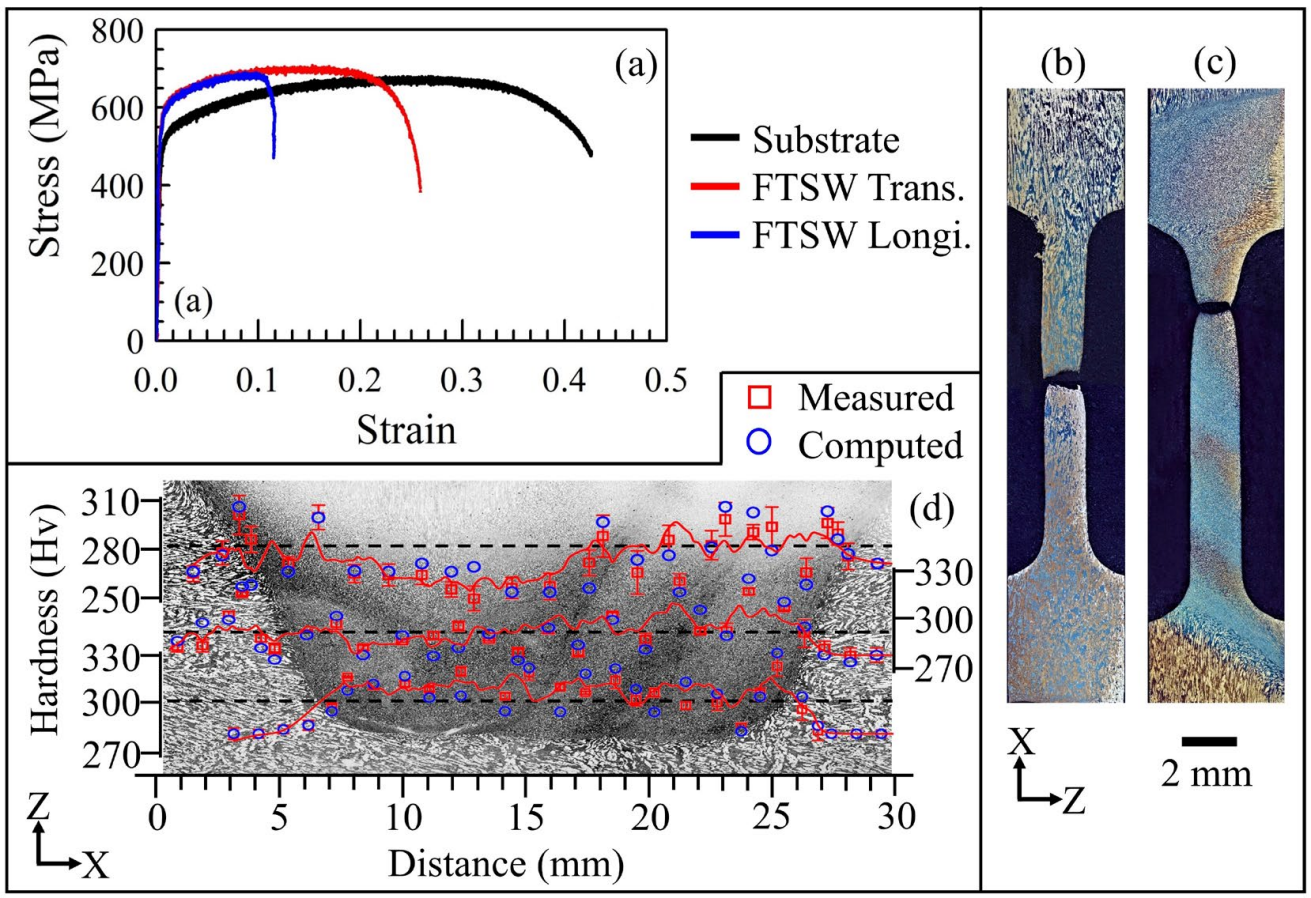
(**a**) Stress–strain responses of substrate and FTSW joint. Macrographs of the fractured tensile testing specimens (**b**) substrate, and (**c**) longitudinal. (**d**) The hardness distribution at three different depths. The longitudinal specimen in Fig (**c**) contained three inserts within the gauge length of the specimen. For tensile testing coupons, etching was done after the tests. Microhardness measurements were carried out without etching, and then a macrograph was produced separately (and shown behind the microhardness data in this figure).

Figure 6a shows the experimentally measured stress–strain behaviour of substrate and FTSW joint. The substrate exhibited nearly 680 MPa of tensile strength and 42.63% elongation as highlighted in Fig. 6a. The transverse specimen from the welded region exhibited a 4.3% rise in tensile strength with an expense of nearly 39.3% drop in elongation in comparison to that of the substrate. The longitudinal specimen from the joint showed a 72.7% drop in elongation with a nominal rise of around 1.7% in tensile strength in comparison to that of the substrate. The significant reduction in the elongation of the longitudinal specimen is attributed to a metallurgical notch, which refers to a specific microstructural feature formed near the central region of the insert due the nearly zero angular velocity at this region, promoting a modification in the orientation of microstructure. Overall, a grain size reduction of the austenite and ferrite phases led to slightly higher tensile strength than that of the as-received material. Figure 6b,c show respectively the fractured substrate specimen and the longitudinal specimen from the welded region. The fracture location in Fig. 6c thus indicates the presence of a metallurgical notch.

Figure 6d shows the computed and corresponding measured hardness distribution at three different heights from the substrate top surface [refer Section 2]. The average computed and corresponding measured hardness values along the topmost line near to the substrate surface are found to be around 303 ± 4 HV and 302 ± 5 HV, respectively. Further, the hardness values at the top line found to scatter within a band 280 ± 20 HV. Similarly, the computed and measured hardness distribution near the middle line found to vary within a narrow range of 290 ± 25 HV. Likewise, the hardness distribution near the bottom line is found to vary within 300 ± 20 HV. The consistent rise in base level hardness from 280 to 300 HV from top to bottom levels is attributed to the corresponding gradual rise in ferrite phase % from substrate top to the insert bottom (Fig. 5b,d). In contrast, the hardness distribution near the top surface is nearly close to the unaffected substrate due to the almost equal phase proportion (Fig. 5d). Overall, a grain size reduction of the ferrite and austenite phases due to FSTW is responsible for a slight improvement in microhardness at certain measurement locations. Finally, the computed hardness distribution corroborates well with the corresponding measured values (Fig. 5).

In summary, a novel numerical heat conduction model is reported to compute the transient temperature field for FTSW of DSS 2205 that involves joining of multiple inserts into a substrate to conceal a longitudinal crack. The frictional heat generation due to introduction of each insert is estimated in a unique manner. The computed thermal cycles are found to be in fair agreement with the measured results. The computed thermal cycles are used further to estimate the distribution of hardness following Johnson–Mehl–Avrami (JMA) relation. A detailed experimental investigation is undertaken to realize the joint structure and properties.

## Conclusions

A systematic coupled experimental and numerical investigation on friction taper stitch welding (FTSW) of DSS 2205 is carried out in the current investigation. Following are the main conclusions.

- The model calculations indicate the occurrence of the peak temperature of ~ 1537 K, which is ~ 93% of the solidus temperature of DSS 2205 at the interface between the insert tip and the substrate crack-hole bottom. The temperature along the side wall of the crack-hole remains little, smaller.
- The temperature field is longitudinally asymmetric around the insert axis as individual inserts are introduced and welded one-by-one along the length of the crack. The last insert to close the crack is likely to experience a near symmetric frictional heating all around its wall interface.
- The computed thermal cycles from the numerical model can be used to realize the influence of process conditions on the joint structure and properties.
- A marginally higher hardness of around 302 HV along the insert hole interface is observed as compared to that in the substrate (280 HV) due to the fine-grained microstructure with an acceptable phase proportion. The estimated hardness distribution from the computed thermal cycles results were in good agreement with the corresponding experimentally measured results.

### Supplementary Information

Supplementary Information.

## Data Availability

All data generated or analyzed during this study are included in this published article and its supplementary information files.
