# Supplementary material for: Friction taper stitch welding of a duplex stainless steel
Source: Sci Rep. 2023 Dec 4;13:21354. doi: 10.1038/s41598-023-48754-6 (PMC10696042; doi:10.1038/s41598-023-48754-6)
Supplement: Supplementary file 1 — Supplementary Information. [file 41598_2023_48754_MOESM1_ESM.docx]

**Friction taper stitch welding of a duplex stainless steel**

Ram Rapaka ^1^, Cleber Rodrigo de Lima Lessa^2^, Guilherme Vieira Braga Lemos^3, 4^, Arlan Pacheco Figueiredo^2^, Buchibabu Vicharapu^1^, Thomas Clarke^4^, Amitava De^5^

*^1^ Indian Institute of Technology Palakkad, Kerala, India.*

*^2^ Federal Institute of Rio Grande do Sul (IFRS), R. Avelino Antônio de Souza 1730, Caxias do Sul 95043‑700, Brazil.*

*^3^ Federal University of Santa Maria (UFSM), Rod. Taufik Germano, 3013, Cachoeira do Sul 96503‑205, Brazil*

*^4^ Physical Metallurgy Laboratory (LAMEF) - PPGE3M/UFRGS, Porto Alegre, Brazil*

*^5^ Indian Institute of Technology Bombay, Mumbai, India.*

**corresponding author (guilherme.lemos@ufsm.br)*

**Supplementary Information Includes**

Appendix-1

Appendix-2

Supplementary Figure A1

Supplementary Table A1

Supplementary References

**Appendix-1**

Figure A1 represents a schematic view of the progressive filling of a crack-hole in the substrate by plasticized insert material. For example, Fig. A1 indicates the plasticization of the insert until a height of H_I_ that fills up the annulus clearance region between the insert and the crack-hole in the substrate until a height of H_C_. Considering the corresponding volumes of plasticized insert material and the filled-up annulus region between the insert and the crack-hole in the substrate as V_I_ and V_C_, the heights H_I_ and H_C_ can be written as :

$H_{I}=\frac{3*V_{I}}{\pi(R_{I}^{2}+R_{I}R_{I+1}+R_{I+1}^{2})}$ (A.1)

$H_{C}=\frac{3*V_{C}}{\pi\left\{ (R_{C}^{2}+R_{C}R_{C+1}+R_{C+1}^{2})-(R_{I}^{2}+R_{I}R_{NI+1}+R_{NI+1}^{2}) \right\}}$ (A.2)

where R_I_ is the radius of the tip of the insert, R_I+1_ is the radius of the insert at a height H_I_, and R_C_ and R_C+1_ are the radii of the insert and the hole, respectively at a height H_C_. Hence, $R_{I+1}=R_{I}+H_{I}\tan(\alpha/{2)}$ and $R_{C+1}=R_{C}+H_{C}\tan(\beta/{2)}$, where $\alpha and \beta$are included angles of insert and hole, respectively. The radius of insert at height *H_C_* is $R_{NI+1}=R_{I}+H_{C}\tan(\alpha/{2)}$.


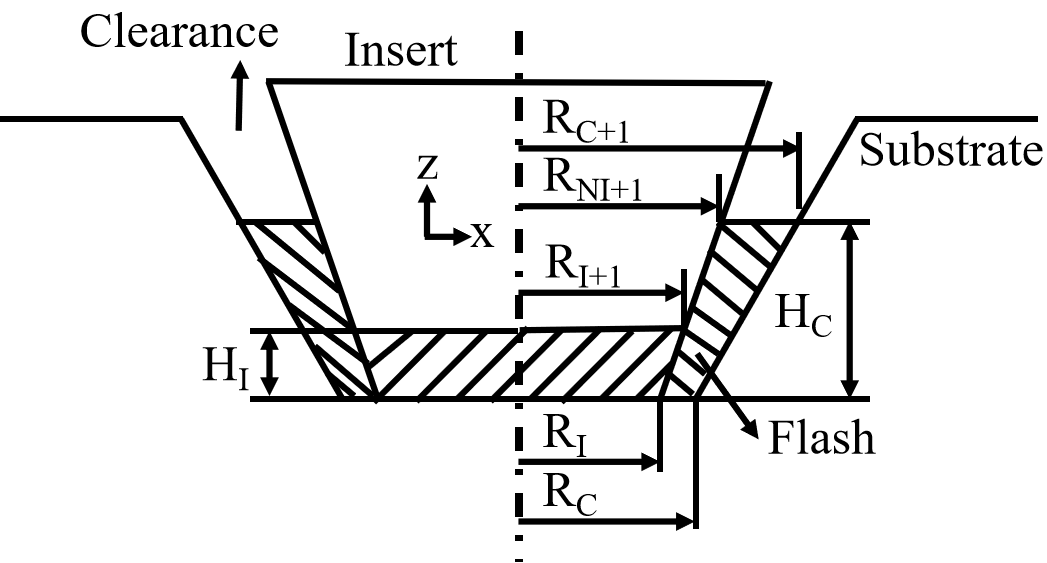


**Figure A1** Schematic depicting the estimation of filling height at insert - hole clearance

**Appendix-2**

The JMA model is applied to the thermal cycle by dividing it into multiple small isothermal time steps ($\Delta t$) and applying the additive principle as

$\sum\frac{\Delta t}{t}=\frac{\Delta t_{1}}{t_{1}}+\frac{\Delta t_{2}}{t_{2}}+...+\frac{\Delta t_{a}}{t_{a}}=1$ (A1)

Equ. (3) in section 3 is modified further to estimate iso-thermal aging time (t_a_)

$t_{a}=\left[ \frac{ln(1-\frac{H_{o}-H}{H_{o}-H_{\infty}})}{-{\beta_{o}e}^{-\frac{Q}{\mathrm{RT}}}} \right]^{\frac{1}{n}}$ (A2)

The JMA model for DSS2205 starts from a temperature of 1075K at which ferrite-austenite transformation takes places [1]. Figure A1 shows two thermal cycles at 0.87 mm, away from the interface of the second insert located inside the first insert. The first thermal cycle has two potential heat cycles that have temperatures above 1075K that are taking part in phase transformation there by hardness variation.

Table A1 presents the sample calculations for the hardness prediction for first potential thermal cycle highlighted in Fig. 4(d). Sum of all Δt/t values shown in Table 1 is iteratively solved to satisfy the equ. (A1) by varying the hardness (H). For example, the sum of all Δt/t values becomes 1 at a hardness value (H) of 319.8 HV at the end of the first cooling cycle. As the peak temperature in the second thermal cycle exceeds the phase transformation temperature, a similar procedure is repeated by assuming H_0_ as 319.8 HV yielding a final value of 290.67 HV.

**Table A1** Hardness calculation for a computed thermal shown in Fig. A1

| Time (s) | Temperature (K) | t | $\Delta t$ | $\Delta t/t$ |
| --- | --- | --- | --- | --- |
| 5.83324 | 1081.57 | 1790.31 | N/A | N/A |
| 5.93324 | 1100.88 | 635.88 | 0.1 | 0.00 |
| ….. | ….. | ….. | ….. | ….. |
| 7.5698 | 1239.47 | 0.97 | 0.0243 | 0.02 |
| ….. | ….. | ….. | ….. | ….. |
| 8.05048 | 1166.51 | 24.36 | 0.18452 | 0.01 |
| 8.32728 | 1111.27 | 369.78 | 0.2768 | 0.00 |

**References**

[1] Tucker, J.D., Miller, M.K., Young, G.A., (2015). Assessment of thermal embrittlement in duplex stainless steels 2003 and 2205 for nuclear power applications. *Acta Mater.*, 87, 15-24.
